# Examining the Suitability of the List of Indicators Describing Age-Related Typical Support Needs

**DOI:** 10.3390/ijerph18020764

**Published:** 2021-01-18

**Authors:** Antonio M. Amor, Miguel A. Verdugo, Benito Arias, María Fernández, Alba Aza

**Affiliations:** 1 Faculty of Psychology, Institute for Community Inclusion, University of Salamanca, Avda. De la Merced, 109-131, 37005 Salamanca, Spain; aamor@usal.es (A.M.A.); verdugo@usal.es (M.A.V.); azhernandez@usal.es (A.A.); 2 Faculty of Education and Social Work, Institute for Community Inclusion, University of Valladolid, Paseo de Belén, 1, 47001 Valladolid, Spain; barias@psi.uva.es

**Keywords:** intellectual disability, developmental disability, inclusive education, support needs assessment, training material, students with special educational needs, students with intellectual disability, students with disabilities

## Abstract

The list of indicators is a form of training material used for the Supports Intensity Scale—Children’s version (SIS-C). It is aimed at helping interviewers distinguishing between extraordinary and age-related typical support needs in children with intellectual and developmental disabilities (IDD) when implementing the SIS-C, and thus improve supports planning. The aim of this study is to adapt and test the list of indicators’ content validity and rating scale’s functioning in Spain. A total of 222 general education teachers reported their agreement with each indicator description using a 5-point rating scale. A total of 353 of 366 indicators showed evidence of content validity, whereas analyses on the rating scale highlighted the necessity of subsuming one of the scale categories within another. The need for developing research-based training materials to develop training programs on the use of the SIS-C to support decision-making concerning supports planning with students with IDD, the relevance of using the latest methodological approaches available when required, and future lines of research are discussed.

## 1. Introduction

Inclusive education for students with intellectual and developmental disabilities (IDD) is a major challenge in education policy agendas. As a movement, since the United Nations Convention on the Rights of Persons with Disabilities (UNCRPD) and its facultative protocol were passed [1], inclusive education has evolved from a principle guiding education to a right to be guaranteed for all students. The UNCRPD embodies the right to inclusive education in its Article 24, which stipulates that states parties must ensure an inclusive education system at all levels and lifelong learning for all students directed to guaranteeing their access, participation, learning and development to their fullest potential [2,3,4].

More than a decade since the UNCRPD was passed, students with IDD still experience low inclusion rates [5,6]. To reverse this trend and support their inclusion, several scholars (e.g., [3,6,7]) have proposed the adoption of the supports paradigm within general education contexts, an approach developed over decades of research and practice in the field of IDD [8,9]. The supports paradigm to support the inclusion of these students is important because it provides a renewed view of IDD and of the students who have the condition [2,10], a work methodology that focuses on understanding the globality of these students and their holistic support needs in education contexts and activities [5,6,11] and tools that facilitate the implementation of this conceptual and applied framework [12].

The supports paradigm is embedded in a social-ecological approach and strengths-based perspective [13]. Through the social-ecological approach, IDD is conceptualized as a mismatch between personal competencies and environmental demands, defined by the contexts of participation and age- and culturally-valued activities to develop in such contexts [14]. This misfit originates supports needs, defined as a “psychological construct referring to the pattern and intensity of supports necessary for a person to participate in activities linked with normative human functioning” [13] (p. 135). By stressing the interaction “person × environment”, the essential characteristic of the supports paradigm is that all people will present support needs since all people experience mismatches in certain situations and moments of their lives. The key is that the support needs of persons with IDD are extraordinary and extend beyond what most typically functioning people require to participate in the same contexts and activities [15,16].

Applying this paradigm to education means understanding students with IDD as learners who experience mismatches between their personal competencies and the environmental demands posed by education contexts and activities [3,6,10,11]. These demands are defined by what is expected from students with IDD in the classroom (i.e., in relation to access to and learning from the general education curriculum), at school (e.g., social activities or self-determination), and in the community (e.g., school trips). Hence, given that education contexts go beyond the classroom and that education activities are linked not only to learning, the supports paradigm claims that students with IDD may present holistic support needs—not only those related to learning from the curriculum—that will influence their access, participation, learning and development [4].

In fact, in opposition to education perspectives that understand students with IDD as having significant limitations in intellectual functioning and adaptive behavior, the supports paradigm shifts the focus towards the current functioning of the students with IDD and the extraordinary supports they require to access, participate and learn in the same activities and contexts than their same-age typically-developing peers [2,7]. Along with this social-ecological approach, the supports paradigm stands on a strengths-based perspective that assumes that, along with their extraordinary support needs, students with IDD have strengths to build upon, and this should be understood on the same basis as their support needs. Moreover, students with IDD should be the causal agent over the supports they receive so that they take an active role in the definition of vital goals—including those pertaining to their learning—and in the vision of the future used to determine their strengths and support needs [6].

An active role by students with IDD and respect for their self-determination in the definition of their goals, as well as understanding of their strengths and support needs in education contexts and activities, are the starting points from which to provide systems of supports aimed at covering their needs, developing their strengths, improving their functioning and enabling them to achieve personal outcomes aligned with the goals of access, participation, learning and maximum development [3,4,7]. Supports are the resources and strategies to bridge the gap experienced by these students, always bearing in mind the need to maximize “*student x environment interaction*” rather than focusing on rehabilitating the student with IDD [14].

Derived from this view, the supports paradigm brings to education systems the “support needs assessment and planning process” [2,6,7]. This process synthesizes the knowledge on the student learning and vital goals, the information about his or her global support needs and strengths, the available resources and supports, the people responsible for providing the supports, and the contexts and activities in which these supports will be put in place, using personalized educational plans (PEPs) that systematically address the goals of access, participation, learning and development of the student with IDD [4,7,11]. Effective implementation of the supports paradigm in education requires measuring support needs [2,7,11]. Only in this way will it be possible to accurately identify the pattern and intensity of the supports and resources required by students with IDD for them to successfully participate in general education activities and contexts with their typically functioning peers. The importance of the support needs construct in applying this paradigm has motivated the development of tools for its measurement. Although different approaches to measuring support needs exist, efforts are being made to develop standardized measures of the extraordinary support needs of persons with IDD based on the supports paradigm [15,16]. The advances in the measurement of extraordinary support needs have been evident [17], but performing a support needs assessment with psychometric instruments poses challenges that are yet to be addressed.

One challenge relates to the nuances in the support needs construct in the case of children. In childhood, support needs are strongly correlated with age. Hence, children (with and without IDD) present higher levels of support needs the younger they are, and as they age, their support needs decrease [16,18]. Therefore, a practical problem concerning support needs assessment for students with IDD is to distinguish whether the support needs experienced by a child with IDD are linked to his or her age (i.e., age-related typical support needs—support needs that typically-developing same-age peers also possess) or whether, on the contrary, they are extraordinary (i.e., connected to the IDD). This challenge has direct implications for the inclusion of students with IDD: although the goal of support needs assessment is to meet the unique needs of each student, the resources and strategies available for this are limited, especially in education contexts given the cuts in education that hinder quality education for all. Therefore, knowing the areas where students with IDD have extraordinary support needs is a pressing challenge for efficient supports planning that lessens the impact of the economic cuts and brings the supports paradigm—and its implications—closer within schools [15,16]

This age-related concern has been considered in the development of the Supports Intensity Scale—Children’s Version (SIS-C) [12], the first standardized support needs assessment measure for children with IDD. The SIS-C is designed to assess extraordinary support needs in children with IDD aged 5 to 16 years in order to provide the personalized supports they require to access and participate in key activities of their daily living contexts (e.g., school or neighborhood). Because support needs would be confounded by children’s age, Thompson et al. [12] stratified the standardization sample to develop norms according to age cohorts (i.e., 5–6, 7–8, 9–10, 11–12, 13–14 and 15–16 years) and levels of intellectual functioning within each age cohort [18].

The SIS-C is organized into two sections: Part I, Exceptional Medical and Behavioral Needs, and Part II, the Support Needs Scale. Part II focuses on support needs assessment in 61 daily life activities across seven domains: home life (HLA), community and neighborhood (CNA), school participation (SPA), school learning (SLA), health and safety (HSA), social activities (SA) and advocacy (AA). To determine extraordinary support needs, each activity is rated across three dimensions, each one following a five-point Likert rating scale: type of support (0 = none; 1 = monitoring; 2 = verbal/gestural prompting; 3 = partial physical assistance; 4 = full physical assistance), frequency of support (0 = negligible; 1 = infrequently; 2 = frequently; 3 = very frequently; 4 = always) and daily support time (0 = none; 1 = less than 30 min; 2 = 30 min to less than 2 h; 3 = 2 h to less than 4 h; 4 = 4 h or more). The tool can be used to calculate a “support needs index”, which provides information on the point along the extraordinary support needs continuum where children with IDD are, and a “support needs profile”, which yields information on the pattern and intensity of the extraordinary supports required by children with IDD across the seven domains measured. The SIS-C is administered by a qualified interviewer (often a school psychologist or another member of a psycho-pedagogical team with expertise in psychological interview skills) through a structured interview with at least two respondents. Observers reporting the support needs of a child with IDD must know the child well and must have recently observed the child in different contexts [12]. Implementing the SIS-C requires the interviewer to have completed at least a four-year degree and to have received training in the foundations of the supports paradigm and in the SIS-C goal. Moreover, the interviewer must have good interview-related skills (e.g., to create a good rapport with the observers, make explicit their fears and expectations, explain to them clearly that the goal of the tool is to measure support needs and not performance or reformulate and share with the observers the information they provide to be sure that everybody has the same understanding about the support needs of the child with IDD [15]). This tool has already been implemented to expand inclusive opportunities in students with IDD within general education contexts through the development of PEPs that, consistently with the supports paradigm, support these students in key areas of their lives, thus contributing to their educational and social inclusion (e.g., [7,11]).

The SIS-C is being internationally adapted and validated, and several studies have provided evidence of SIS-C’s psychometric properties (for detailed information, see [17]). One country that has been particularly involved in SIS-C validation is Spain since it uses two versions of the tool: the SIS-C Spanish and SIS-C Catalan translations [17]. Currently, much importance is being given in Spain to the use of the SIS-C for a support needs assessment and planning process that overcomes attention to diversity measures that remain anchored in deficit-based perspectives, often neglecting a global view of the students’ needs [2,19], which has led to systematic segregation of students with IDD [20].

However, using the SIS-C in practice necessitates addressing the aforementioned challenge concerning the nature of the support needs of children with IDD. Despite the efforts of SIS-C research to illuminate the distinction between extraordinary versus age-related typical support needs (e.g., [16]), this concern involves decision-making by the interviewer—the person who implements and scores the SIS-C. In this respect, when implementing the SIS-C, the interviewer is the first person to face the challenge of discerning the nature of the support needs of a child with IDD based on the information reported by observers. Hence, the interviewer’s knowledge on this issue may influence how he or she interprets the information reported by the observers and decides on the type of support needs of the child, which, in turn, will influence the allocation of resources and supports to cover the needs of the student with IDD.

Given the importance of training for implementing the SIS-C, the American Association on Intellectual and Developmental Disabilities (AAIDD), the SIS-C launcher, has developed and distributed different training materials to the countries participating in SIS-C validation. These materials aim to train interviewers in implementing and using the SIS-C. To help identify the type of support needs of a child with IDD, the AAIDD developed a list of indicators (hereafter, list of indicators) based on a teachers’ survey. The list of indicators describes age-related typical support needs through examples representing the probable support needs that typically-developing children might have for the same activities, domains and age cohorts used in the SIS-C (given that typically-developing children are expected to present only these support needs and no extraordinary ones). Through these descriptions, this training material seeks to support interviewers by providing qualitative information about the age-related typical support needs for each SIS-C item and thus help them make decisions on the nature of support needs of a child with IDD based on information provided by the observers [21].

Implementing the SIS-C in Spain to develop an efficient support needs assessment and planning strategies requires addressing the practical challenge of discerning the nature of support needs in children with IDD. In this respect, the availability of the Spanish SIS-C task force of the list of indicators may help interviewers address this challenge with the descriptions provided by the list. However, this list remains unexplored in Spain; hence, it is necessary to adapt it and test its appropriateness in this context prior to using it to train interviewers. Considering this requirement, the purpose of this study is twofold: to present the translation and adaptation of the list of indicators in Spain and to furnish evidence of its appropriateness. The research questions guiding the analyses of the appropriateness of the list of indicators are:

- Can the indicators included in the list of indicators be considered valid sources for accurate descriptions of age-related typical support needs for the same activities, domains and age cohorts as those used in the SIS-C in Spain?
- Is the list of indicators an effective survey for collecting teachers’ subjective impressions of age-related typical support needs in the Spanish context (i.e., can the appropriateness of the indicators be ascertained after analyzing how the information used to determine their content validity has been gathered)?

## 2. Materials and Methods

### 2.1. Participants

A total of 222 teachers with a mean age of 40.82 years (SD = 9.59, range 22–63) and an experience of 16.66 years (SD = 9.73, range 1–40) were selected following an incidental sampling method. To participate in the study, teachers were required to work in the general education context for at least three months, either in the stage of primary (from 6 to 12 years old in Spain) or in secondary education (from 12 to 16 years old). Teachers were consulted as experts on age-related typical support needs owing to their daily experience with typically-developing students and given that the development of the list of indicators in the original context was based on a teachers’ survey [21]. Table 1 summarizes participants’ information.

### 2.2. Instrument

The list of indicators is SIS-C training material based on a teachers’ survey [21]. It aims to help interviewers administer and use the SIS-C. Given the importance of age for determining the support needs of children, six versions of the list of indicators corresponding to the SIS-C age cohorts were created (i.e., 5–6, 7–8, 9–10, 11–12, 13–14 and 15–16 years). Considered across all the cohorts, the list of indicators contains a total of 366 descriptions (61 per age band), which are designed to educate interviewers on age-related typical support needs through exemplary descriptions of the probable support needs that typically-developing children aged 5 to 16 years old might have for each SIS-C item. Through these descriptions, this list is intended to help interviewers to distinguish, based on the information provided by observers, whether the reported support needs are likely extraordinary (i.e., linked to the IDD) or related to age (i.e., age-related typical support needs that typically-developing same-age peers also experience).

Each indicator represents a daily life activity in a given domain for a certain age band, followed by a description of exemplary activities, a description of the possible age-related typical support needs that typically-developing children may have to pursue the corresponding activity and the rating scale categories. Teachers express their agreement with the age-related typical support needs described for each indicator by choosing the category that best describes their opinion using a 5-point Likert-type rating scale. All the indicators translated and adapted can be found in [21]. Table 2 shows an example of SIS-C item of the SPA domain for the 7–8 age band, followed by the description provided by the list of indicators on typical age-related support needs that 7–8-year-old typically-developing children would have to participate in the activity and the rating scale used by teachers to show agreement with the description.

### 2.3. Procedure

Three steps were followed to address the goal of the study: (a) translation and adaptation of the list of indicators, (b) data collection of teachers’ subjective impressions of the indicators’ descriptions and (c) data analysis.

First, the indicators were translated and adapted using Tassé and Craig’s [22] guidelines for effectively adapting items to different contexts from the original context: (a) translation/adaptation, (b) consolidation of translation/adaptation, (c) validation of preliminary translation, (d) revision/adjustments, (e) pilot testing, (f) revision/adjustments and (g) field test validation.

All indicators and the survey’s rating scale options were independently translated by two of the authors who possess accredited English language knowledge. Only certain exemplary activities were changed for cultural reasons (e.g., watching a baseball game became watching a soccer game). Because none of the research team members was an English native, the research team included another step in Tassé and Craig’s [22] guidelines, and the translated indicators were sent to a native English speaker, who translated them back into English. Finally, the entire team ensured that the meaning of the indicators remained unchanged.

Once translated, the indicators were sent to different researchers for feedback and suggestions on improving the indicators. Minor corrections were made, and consequently, the instrument was ready for use. Thereupon, the research team contacted different schools to share the research goal and request teachers’ collaboration.

After schools had agreed to collaborate, the first author visited the schools and organized a two-hour seminar with the teachers who were willing to participate. During the seminar, the author explained the supports paradigm and how to complete the task using the list of indicators. Teachers were required to select the version of the list of indicators that matched the age groups they taught (e.g., a teacher working with 16-year-old students needed to select the 15–16 version) and show their agreement with each indicator description using the rating scale. Additionally, examples were made to help understand the task in practice, and all the doubts were addressed. After the seminar, teachers were given a two-week period to complete the tool and received via email a guide on how to complete the task and contact information for the potential doubts they may have during its completion. Once the instruments were completed, they were collected for data analysis. This research received approval by the ethics committee of the University of Salamanca (resolution available upon request). Further, all procedures were in accordance with the General Data Protection Regulations (Regulation (EU) 2016/69) and the 1964 Helsinki declaration and its amendments.

### 2.4. Data Analysis

A descriptive, cross-sectional study was conducted. Different data analysis strategies were followed to address each research questions

#### 2.4.1. Research Question 1—Content Validity Analysis

Bangdiwala’s weighted statistic for ordinal data (B^W^_N_) and Bangdiwala’s agreement chart [23] was calculated for each indicator to study content validity to determine how well the indicators reflect age-related typical support needs. The B^W^_N_ allows calculating the agreement level among judges (by judges, we refer to the teachers who categorized each indicator using the rating scale) for each indicator to study the judges’ agreement strength. In other words, the study focused not on the agreement between judges, but on the agreement size among judges regarding the indicators to categorize (e.g., a perfect agreement between judges can be found for a category different from agreeing, which would indicate weak evidence of content validity for a given indicator). This statistic expresses agreement strength on a scale from 0 to 1, with 0 indicating the absence of agreement and 1, the strongest agreement possible. Agreement strength can be poor (0.000 to 2.00), weak (0.201 to 0.400), moderate (0.401 to 0.600), good (0.601 to 0.800) and very good (0.801 to 1) [23].

One advantage of the B^W^_N_ is its graphical approach, allowing researchers to represent the distribution of agreement to complement B^W^_N_. Bangdiwala’s agreement chart provides a representation of the agreement among judges based on a contingency table. The chart is built as a square, *n x n*, where *n* is the total sample size. The black squares, each one measuring *nii x nii*, show the observed agreement. The black squares are within larger rectangles; each one sized *ni + x n + i*. These rectangles show the maximum possible agreement, given the marginal totals. Partial agreement is determined by including a weighted contribution from the cells outside the diagonal and is represented in the chart with shaded rectangles, whose sizes are proportional to the sum of the frequencies of the cells [23]. Analyses involving content validity were addressed using the software R v.3.4.2 [24].

#### 2.4.2. Research Question 2—Rating Scale Assessment

The many-facet Rasch measurement (MFRM) model was used to assess the appropriateness of the rating scale used by teachers to show their agreement with the indicators’ descriptions. The MFRM model is commonly used for performances evaluated with subjective ratings (e.g., speaking assessments), permitting researchers to obtain estimates on a common logit scale of the parameters of the components of the facets involved in construct evaluation [25]. In the construct assessments based on judges’ evaluations, such as those used in this study, the importance of judges’ severity or leniency in determining these evaluation scores, as well as the difficulty of the tasks evaluated, has been highlighted, with the judges and tasks being treated as facets of the construct assessment [26].

The indicators of the list of indicators and the teachers were considered facets of construct evaluation along a logit scale representing the “age-related typical support needs” construct (for the rating scale-related analyses and results, the terms “judge” and “item” will substitute “teachers” and “indicators”, given that this jargon is more common regarding the MFRM model). The analysis of the rating scale focused on judges’ assessment of how the rating scale, developed for assessing each item’s accuracy in describing the age-related typical support needs, was useful for the Spanish context. The aim was to explain whether the 5-category rating scale worked properly using a strong logistic model for assessing the quality of tests (the list of indicators is a survey that collects subjective ratings). Nevertheless, prior to positing any explanations on the rating scale’s functioning, it was necessary to ascertain the facets’ adjustment to the MFRM model (depending on the estimates of their parameters on the common scale). To consider the facets as adjusted to the MFRM model, four estimates need to be calculated: SD, separation, strata, and reliability. Items’ adjustment is indicated by high SD, separation > 1, strata > 2 and reliability > 0.80, whereas judges’ adjustment to the models requires low SD and separation, strata < 2 and low reliability [25]. Thus, evidence of the facets’ misfit would add noise, and no interpretation of the rating scale should be undertaken [27]. Hence, to assess evidence of the rating scale’s functioning, it was first necessary to analyze the facets’ adjustment to the model, then assess whether the rating scale was working. To analyze the rating scale’s adjustment, the Rasch–Andrich thresholds (*τ*) were calculated. In the case of a polytomous rating scale (as in the teachers’ survey used in this study), *τ* are understood as local dichotomies between adjacent Likert-scale steps [26]. The rating scale’s fit to the MFRM model is possible only if the *τ* values exhibit a rising progression or monotonic order [25].

The MFRM model is iterative. Thus, if the data (facets and/or rating scale) evidence a poor model adjustment, researchers can test where the problem may be (e.g., if the problem involves judges’ facets, extreme cases can be removed) and conduct additional estimations to test whether data adjustment to the model is possible [25]. Information on the facet and rating scale adjustment to the MFRM model, the facets’ distributions along the common logic scale and the probability curves of the rating scale categories were analyzed and are reported in the results section (see Section 3.2). Facets software v.3.71.3 [27] was used to answer this research question.

## 3. Results

### 3.1. Research Question 1—Content Validity Analyses

The B^W^_N_ and charts were calculated for each indicator within each age cohort, totaling 366 calculations to analyze the content validity of the indicators for describing the age-related typical support needs that typically-developing children may have. Owing to word limits, the B^W^_N_ results for each indicator alongside its representation cannot be shown, but all data (i.e., B^W^_N_ and charts) are available as supplementary material (see Table S1). The percentages of indicators are presented according to the “agreement” range overall and for each age group. The minimum, maximum and mean of agreement size are shown for each domain, considering the age groups. The indicators that did not show content validity are also reported.

Table 3 provides the agreement size ranges (in percentages) for the entire list of indicators across all age cohorts. As shown, the agreement size was very good and good for nearly all indicators (94.54%) and was around the agreement category, thus providing evidence of content validity.

To examine the indicators in-depth, Table 4 summarizes the minimum, maximum and mean of the B^W^_N_, considering domains and age cohorts. The results also indicated high agreement among judges when categorizing activities.

Although results at the indicator level demonstrated content validity for 353 indicators, specifying the indicators that did not show content validity is necessary. For those indicators, either the B^W^_N_ was low, or the agreement chart was not close to the agree category. Table 5 illustrates these indicators alongside Bangdiwala’s agreement charts.

### 3.2. Research Question 2—Rating Scale Analyses

Different iterations were necessary to achieve full data adjustment. The iteration processes are presented with the facet and rating scale estimates. In iteration 4 (where the data fitted the model), a Wright’s map showing the facet (i.e., judges and items) distributions along the common logic scale and the probability curves of the rating scales categories are reported.

#### 3.2.1. Iteration 1

Results on the facets’ adjustment to the MFRM model varied. Items estimates evidenced a good fit (SD_items_ = 0.42; separation_items_ = 3.81; strata_items_ = 5.42; and reliability_items_ = 0.94), whereas judges estimates did not (SD_judges_ = 0.65; separation_judges_ = 3.06; strata_judges_ = 4.41; reliability_judges_ = 0.90). Regarding the rating scale’s adjustment, the data did not fit the model. Although the categories’ average mean values exhibited a rising progression (−1.27; −0.53; −0.06; 0.46; 0.89), the *τ* values did not (−1.70; −2.54; 1.45; 2.80). Upon closer examination of the data, it seemed that the problem lay between *τ1* (−1.70) and *τ2* (−2.54), suggesting, from the logistic model, that category 1 (disagree; students need less support than described) was not the most likely along the continuum [25].

Prior to asserting that this rating scale’s category was invalid, it was necessary to identify the reason for the misfit in the case of both the judges and the rating scale. To be orthodox, the authors decided to first remove judges that did not fit the model and then repeat the analyses to determine whether the judge and rating scale fit was possible. Judges whose outfit values were higher than 3 ZStd and lower than −3 ZStd were removed (see Table 6) because they were considered “extreme” [25].

#### 3.2.2. Iterations 2 and 3

After removing extreme judges, estimations were recalculated for depurating the model fit. Table 7 summarizes the facet and rating scale estimates, identifying extreme judges by their outfit values for each iteration. As shown, the data did not fit the model, and the problem again lay with the judges’ estimates and the transition between τ1 and τ2 (i.e., category 1).

Once the first possibility had been analyzed (i.e., misfit caused by extreme judges) and the data still did not fit the model, it was necessary to determine whether the misfit stemmed from the rating scale being ineffective. Thus, the lack of monotonic order of *τ* values in the three iterations with the problem in the transition between *τ*_1_ and *τ*_2_ indicated that category 1 was not working. It seemed that this category was ambiguous and that judges may have misunderstood it. To test whether the problem was in category 1 based on the *τ* values found, the authors collapsed category 1 within category 0 and then repeated the analyses and tested the facet and rating scale (4 categories now) adjustment to the MFRM model (agree was now in category 1 instead of category 2).

#### 3.2.3. Iteration 4

Two parallel analyses were conducted after collapsing categories: (a) an analysis without the extreme judges and (b) an analysis with all the judges. After collapsing categories, whether the judges previously considered extreme were included in the data pool was inconsequential because all the data fitted the model (see Table 8), indicating that the previous misfit problem did not concern the judges, but the original rating scale.

**Figure 1:**
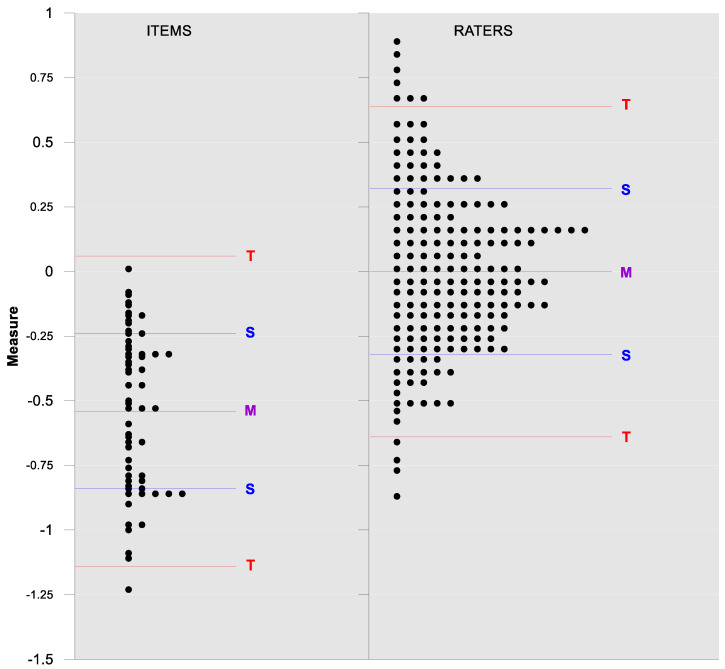
Wright’s map.

Wright’s map and the category probability distribution (without extreme judges; *n* = 181) are presented in this iteration. Wright’s map (Figure 1) represents both the items (based on each item’s difficulty in representing the age-related typical support needs) and the judges (ranked by their severity/leniency when assessing each item) in the logit scale situated in the central axis (positive indicates a “high level” of the construct, while negative indicates a “low level”). The figure describes the facets’ relationships across the logit continuum and communicates important information. First, regarding the logit scale data, the judges’ mean (M = 0.00; SD = 0.32) was slightly higher than the items’ mean (M = −0.54; SD = 0.31). Second, the judges’ spread (−0.87 to 0.89 logits) was also higher than the items” spread (0.01 to −1.23). Third, 78 judges (43.09%) scored above the items’ range, whereas no judge scored below it. Finally, the targeting region in the logits between item difficulty and latent construct presence in judges corresponded to more than half of the participants (56.91%), indicating an acceptable relationship between the facets in the logit scale [25].

**Figure 2:**
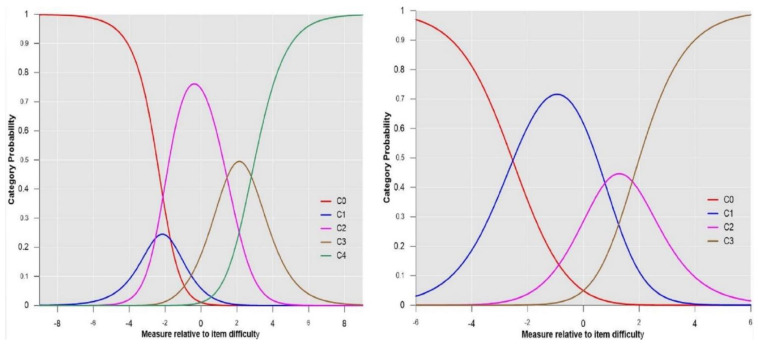
Rating scale category probability curves. The left side represents iteration 1: C0 = strongly disagree; students need far less support than described; C1 = disagree; students need less support than described; C2 = agree; C3 = disagree; students need more support than described; C4 = strongly disagree; students need far more support than described. Right side depicts iteration 4 (with collapsed categories): C0 = less support than described; C1 = agree; C2 = disagree; students need more support than described; C3 = strongly disagree; students need far more support than described.

Figure 2 illustrates the differences between the rating scale categories’ probability curves along the logit continuum with respect to item difficulty in iterations 1 and 4. In iteration 1 (left), category 1 (blue curve) was not the most likely category in the common scale, indicating a lack of functioning of the category. However, in iteration 4 (category 1 collapsed within category 0), all categories worked since each category was the most probable one at some interval of the construct.

## 4. Discussion

This article presents evidence of the appropriateness of SIS-C training material in Spain to support interviewers to discern the nature of the support needs of children with IDD while implementing the SIS-C. Achieving this distinction is critical for efficient support needs assessment and planning that maximizes resource allocation in education to support the inclusion of students with IDD. Content validity analyses of the indicators were conducted, and the rating scale’s appropriateness of the list of indicators was examined.

Regarding the first research question, the B^W^_N_ and Bangdiwala’s agreement charts were calculated for each indicator. Judges (teachers) exhibited strong agreement when categorizing the accurateness of the indicators describing age-related typical support needs. For 353 indicators, the agreement size was high and around the agree category, showing evidence of their content validity. Only 13 of the 366 indicators presented difficulties regarding content validity. These indicators were situated mainly within the 9–10, 13–14, and 15–16 age cohorts in the HSA and AA domains, and professionals tended to consider that greater support was required (i.e., an agreement concerning the category disagree; students need more support than described).

Different explanations may illuminate the results for these indicators. The areas for which the indicators did not function well are related to health, self-determination and social relationships for children aged 9–10 years and adolescents aged 13–16 years. Before further research is undertaken, developmental psychology can provide insights into these results. A constant in human development research is that as people grow and reach certain stages of development, developmental milestones become increasingly complex [28]. Therefore, milestones can identify particular difficulties during adolescence that are due to risk-taking behaviors [29,30] related to the HSA domain and social, cognitive, emotional and behavioral changes and competencies [31,32] linked to interactions with others (which involve the SA and AA domains). Thus, as people grow, they face new challenges that may demand greater support from others, and teachers—perhaps aware of this—have considered that the typical age-related support needs to be described for these activities and domains needed to include descriptions indicating more support.

Concerning the rating scale’s assessment analyses, 222 judges assessed how the rating scale works while assessing indicators (i.e., items) describing age-related typical support needs. These analyses show that category 1 (disagree; students need less support than described) seemed not to have been understood by judges in Spain, as evidenced by data from multiple iterations that tested the facet and rating scale adjustment to the MFRM model. The adjustment was achieved only after collapsing categories, whereas all other categories (including agree) showed a good fit, indicating that the judges understood them.

The results of the rating scale’s analyses highlight the most important finding of this work, particularly when considered alongside the results of content validity. The fact that one of the rating scale’s categories did not work in Spain implies that although evidence on content validity was found for 353 indicators, the indicators should not be used. Determining whether the indicators work is impossible since the rating scale used to gather the information used for testing their content validity did not fit the logistic model. Hence, additional research is required before using the list of indicators to train interviewers in Spain to support the SIS-C use.

The lack of international studies furnishing evidence of the appropriateness of this list of indicators hinders the generation of discussion regarding our findings. Nevertheless, the main finding reported in this study in relation to this material in Spain has important implications for researchers working on SIS-C validation who have access to SIS-C training materials. If training materials associated with the SIS-C (regardless of their purpose) are to be used, then rather than assume they are valid, the appropriateness of those materials must be analyzed. If the gathered evidence suggests that the material requires additional research (as in this case), there is no methodological justification for its use. However, without analyzing these materials, whether their use is justified, cannot be known. Given that interviewers must be qualified to administer the SIS-C [12] and that this qualification is provided through training, offering interviewers training based on materials whose appropriateness is unproven could bias the training. This bias may distort information gathering through SIS-C use, providing a poor basis for support planning, which could hinder the development and inclusion of children with IDD instead of enhancing their opportunities. Hence, the lack of studies that have analyzed SIS-C training materials and the list of indicators is troubling because these indicators are closely related to the use of the SIS-C, a tool intended for international use in areas such as health, social services and education. Thus, additional studies on this topic are required to generate discussion.

Another implication of this work is that the latest available approaches are preferable to address a research concern when necessary. In this study, not only did we conduct analyses of content validity, but also we performed analyses of how the information used for that purpose was gathered (i.e., rating scale’s assessment). In this case, if the information provided by teachers had been used only for content validity analyses, the main finding of this work would have been evidence of content validity for nearly all the indicators. However, as discussed, the MFRM model analyses indicated the need for additional research prior to the use of the list of indicators in Spain.

The present research has several strengths. First, it foregrounds the SIS-C training materials as the object of study. This study is the first to contribute evidence concerning the list of indicators, which aims to help interviewers address challenges concerning SIS-C use, like discerning the nature of support needs in children with IDD. Second, this work offers researchers who have access to SIS-C training materials a methodological framework for gathering evidence on the list of indicators to generate discussion. Third, this work has been parsimonious, and thus the content validity of each indicator was studied. Finally, although the number of participants per age cohort can be considered reduced, to our knowledge, this study is the only work to assess the appropriateness of a survey’s rating scale (i.e., in this case, the list of indicators), adopting the MFRM model using 222 judges.

However, this work also has limitations. First, the study used an incidental sample, which does not assure representativeness and affects the generalizability of results. On the other hand, the number of participants per age cohort was small. Considering this limitation, a bootstrapping strategy was adopted to generate 10,000 different versions of the same data pool. Second, regarding the rating scale’s assessment, the judges were all teachers, so testing the extent to which their expertise influenced the results was impossible. Finally, the list of indicators (training material), the study design (contributing evidence on the list’s appropriateness) and the results (additional research is required before using this material in Spain) highlight limited yet important practical implications of this research.

Thus, although additional research is required before using the list of indicators in Spain, the significance of a valid list of indicators is worth stressing, given its role in supporting the use of the SIS-C to distinguish between extraordinary and age-related typical support needs in children with IDD. In this sense, the importance of training for SIS-C implementation and scoring [12] necessitates the development of training programs with different goals (e.g., discerning the nature of the support needs of children with IDD). The significance of offering evidence concerning the appropriateness of SIS-C training material is that it guarantees an adequate starting point to develop such training programs. Once developed, it will be necessary to investigate the efficacy of the training programs, given their purpose. Thus, this work has an applied relevance on which to base the development of SIS-C training programs.

The limitations highlighted serve as a starting point for future research. Regarding the rating scale’s analyses, although the present authors took the approach followed in the original list of indicators using teachers as participants, participants from different areas (e.g., social work or psychology) should be included, and the ratings provided by them should be compared with those presented in this study to analyze the presence or absence of biases depending on each professional’s expertise. If the data again show that the rating scale is ineffective, then redefining the categories would be necessary, as the MFRM model shows. Thereafter, analyses of the content validity of the indicators should be conducted. If the data suggest that certain indicators do not show evidence of content validity, a qualitative study should be conducted addressing which age-related typical support needs, in the participants’ opinions, would be appropriate to pursue the activities corresponding to the indicator, to improve the indicators’ accurateness.

Finally, the development of a training program aimed at enhancing the scoring of the SIS-C must have a clear end in mind: to offer to schools and psycho-pedagogical teams support for a more efficient supports planning to shorten the distance between the competencies of students with IDD and the environmental demands they face in education contexts and activities to promote their access, participation, learning, and development to their fullest potential. Only through this can be built a strong and inclusive society [33] where all people, no matter their personal or social conditions, contribute on an equal foot with others, as citizens whose rights are guaranteed [1].

## 5. Conclusions

This work is the first to set evidence on the appropriateness of the list of indicators, a training material based on a teachers’ survey that has been developed to support SIS-C interviewers in discerning the nature of the support needs of children with IDD when implementing the SIS-C. To furnish evidence on its appropriateness, taking into consideration the opinions yielded by 222 teachers, the content validity of the 366 indicators has been studied, and the rating scale appropriateness to catch teachers’ subjective impressions when rating the accurateness of the indicators was examined. Although evidence on content validity has been found for 353 indicators, the results concerning the rating scale, with category 1 (disagree; students need less support than described) showing a lack of adjustment to the MFRM model, prevent the use of the list of indicators to train interviewers until further research is undertaken. This work also has provided directions for further research that addresses the limitations found in the study.

## Figures and Tables

**Table 1:** Participants’ sociodemographic characteristics.

Variable	*n*	%	Variable	*n*	%
Gender			Schooling		
Male	69	31.1	Private school	115	51.8
Female	147	66.22	Public School	107	48.2
Missing	6	2.68			
Age Cohort			Autonomous community		
5–6	37	16.67	Castile and Leon	88	39.64
7–8	35	15.76	Extremadura	102	45.95
9–10	35	15.76	Castile-La Mancha	3	1.35
11–12	37	16.67	Community of Valencia	28	12.61
13–14	43	19.38	Cantabria	1	0.45
15–16	35	15.76			

**Table 2:** Supports Intensity Scale—Children’s version (SIS-C) item, related indicator describing age-related typical support needs and rating scale used to show agreement with the latter (7–8 years old version).

School Participation Activities: Item 2 (7–8-Years-Olds Version)	Indicator Describing Typical Age-Related Support Needs that 7–8-Year-Old Typically-Developing Children Might Present
Participating in activities in common school areas (e.g., playground, hallways, cafeteria)	Most 7–8-year-old typically-developing children need verbal or visual support from one or two educators that provide prompts to groups of children outside the classroom, such as in hallways, playground and cafeterias. Once they are familiar with procedures and routines, typically-developing children only need personalized instructions occasionally and, hardly ever, need support in social interactions with others. Individual support is minimal, less than 30 min a day
**Please, show your agreement with the indicator’s description by choosing one of the following options**	
	□ 0 = strongly disagree; students need far less support than described □ 1 = disagree; students need less support than described □ 2 = agree □ 3 = disagree; students need more support than described □ 4 = strongly disagree; students need far more support than described

**Table 3:** Judges’ agreement size (age cohorts).

Age Cohort	B^W^_N_ Ranges (% of Indicators)				
	Poor 0.000–0.200	Weak 0.201–0.400	Moderate 0.401–0.600	Good 0.601–0.800	Very Good 0.801–1
5–6	0	0	4.92	14.75	80.33
7–8	0	1.64	3.28	24.59	70.49
9–10	0	0	0	44.26	55.74
11–12	0	0	3.27	36.07	60.66
13–14	0	3.28	11.47	21.31	63.94
15–16	0	1.64	3.28	36.07	59.01
General	0	1.09	4.37	29.51	65.03

**Table 4:** Agreement size among judges (domains and age cohorts).

Age Cohort	5–6			7–8			9–10			11–12			13–14			15–16		
	(B^W^_N_)			(B^W^_N_)			(B^W^_N_)			(B^W^_N_)			(B^W^_N_)			(B^W^_N_)		
Domain	m	M	*M*	m	M	*M*	m	M	*M*	m	M	*M*	m	M	*M*	m	M	*M*
HLA	0.75	0.92	0.86	0.49	0.89	0.81	0.64	1	0.77	0.65	0.89	0.78	0.44	0.96	0.82	0.54	0.95	0.84
CNA	0.84	0.92	0.88	0.65	0.87	0.81	0.64	0.83	0.77	0.58	0.85	0.76	0.71	0.95	0.87	0.76	0.87	0.79
SPA	0.72	0.95	0.83	0.79	0.88	0.84	0.73	0.91	0.81	0.76	0.94	0.84	0.73	0.92	0.77	0.57	0.92	0.84
SLA	0.82	0.92	0.87	0.39	0.92	0.77	0.74	0.89	0.83	0.76	0.91	0.82	0.31	0.93	0.80	0.70	1	0.84
HSA	0.45	0.97	0.71	0.50	0.97	0.83	0.65	0.91	0.84	0.49	0.90	0.73	0.31	0.88	0.67	0.22	0.89	0.71
SA	0.72	0.94	0.87	0.62	0.95	0.84	0.70	0.95	0.85	0.82	0.87	0.85	0.52	0.89	0.75	0.71	0.86	0.79
AA	0.45	0.96	0.80	0.62	0.96	0.85	0.76	0.89	0.81	0.76	0.92	0.85	0.57	0.91	0.81	0.66	0.87	0.79

Note. B^W^_N_ = Bangdiwala’s weighted statistic; m = minimum; M = maximum; *M* = mean; HLA = home life; CNA = community and neighborhood; SPA = school participation; SLA = school learning; HAS = health and safety; SA = social activities; AA = advocacy.

**Table 5:** Indicators showing weak evidence on content validity (age cohorts and domains).

Domain/Indicator (Age Cohort)	B^W^_N_	Chart	Domain/Indicator (Age Cohort)	B^W^_N_	Chart
SLA/04 (7–8)	0.72	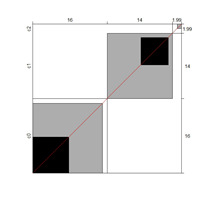	HSA/07 (13–14)	0.68	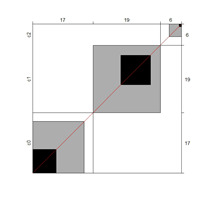
CNA/07 (9–10)	0.71	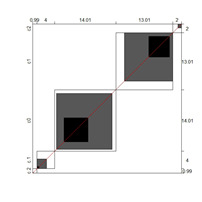	HSA/08 (13–14)	0.87	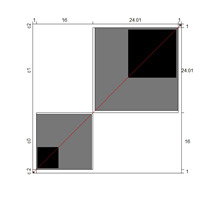
SLA/03 (9–10)	0.78	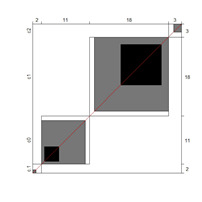	AA/03 (13–14)	0.78	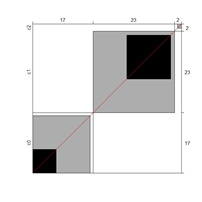
AA/02 (9–10)	0.78	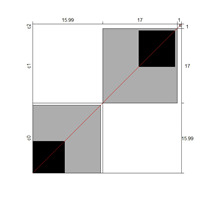	HSA/08 (15–16)	0.22	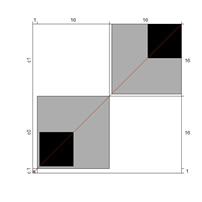
AA/09 (9–10)	0.76	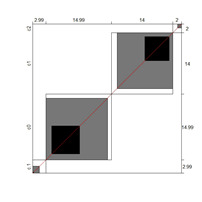	SA/02 (15–16)	0.75	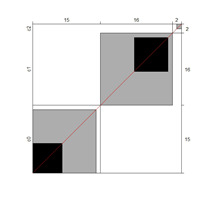
CNA/05 (13–14)	0.71	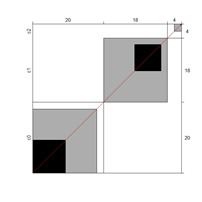	AA/04 (15–16)	0.85	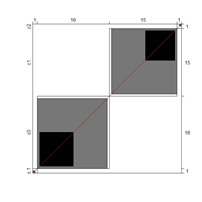
HSA/06 (13–14)	0.68	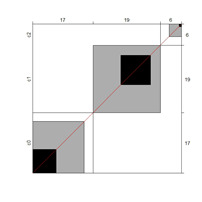			

Note. B^W^_N_ = Bangdiwala’s weighted statistic; HLA = home life; CNA = community and neighborhood; SPA = school participation; SLA = school learning; HSA = health and safety; SA = social activities; AA = advocacy. For these activities, B^W^_N_ and charts indicate that, except for HSA/08 (15–16) indicator, the agreement was distributed between categories 2 (agree) and 3 (disagree; students need more support than described).

**Table 6:** Maladjusted judges to many-facet Rasch measurement (MFRM) model (*n* = 37).

Judge	Logit (SE) Severity/Leniency	Outfit (ZStd)	Judge	Logit (SE) Severity/Leniency	Outfit (ZStd)
221	−1.21 (0.20)	5.7	032	0.29 (0.22)	−5.5
008	−1.17 (0.20)	3.5	052	0.29 (0.22)	−4.8
022	−1.13 (0.20)	3.2	157	0.29 (0.22)	−4.3
140	−1.09 (0.20)	3.3	018	0.34 (0.22)	−6.6
219	−1.05 (0.20)	4.2	016	0.38 (0.21)	−6.0
056	−1.01 (0.20)	5.8	019	0.43 (0.21)	−5.6
220	−1.01 (0.20)	3.9	020	0.43 (0.21)	−4.4
207	−0.89 (0.20)	4.2	217	0.43 (0.21)	−4.1
097	−0.72 (0.21)	3.4	033	0.47 (0.21)	−4.9
096	−0.67 (0.21)	3.1	036	0.47 (0.21)	−2.5
208	−0.67 (0.21)	3.7	072	0.47 (0.21)	−3.2
201	−0.39 (0.22)	3.3	069	0.52 (0.21)	−4.9
172	−0.25 (0.22)	3.1	035	0.56 (0.21)	−4.6
118	0.00 (0.22)	3.1	062	1.48 (0.16)	4.00
039	0.05 (0.22)	6.9	004	1.73 (0.15)	3.7
030	0.10 (0.22)	−3.4	109	1.78 (0.15)	3.2
013	0.20 (0.22)	−3.9	206	2.71 (0.17)	6.2
049	0.20 (0.22)	−3.5	210	2.83 (0.17)	7.1
121	0.20 (0.22)	−3.7			

Note. SE = standard error.

**Table 7:** Data fit (iterations 2 and 3).

	Facets								Maladjusted Judges			Rating Scale’s Categories	
	Items				Judges								
	SD	Sep.	Strat.	Rel.	SD	Sep.	Strat.	Rel.	Judge	Logit (*SE*) Severity/Leniency	Outfit (ZStd)	Avg. Meas.	*τ*
Iteration 1	0.44	3.54	5.05	0.93	0.59	2.70	3.94	0.88	174	−0.44 (0.22)	3.3	0 = −0.97 1 = −0.58 2 = −0.02 3 = 0.50 4 = 0.85	*τ*_1_ = −2.02 *τ*_2_ = −2.49 *τ*_3_ = 1.46 *τ*_4_ = 3.04
									095	−0.34 (0.23)	3.2		
									101	−0.23 (0.23)	3.3		
									139	1.08 (0.19)	3.1		
Iteration 3	0.44	3.47	4.96	0.92	0.61	2.73	3.98	0.88	NONE			0 = −1.00 1 = −0.60 2 = −0.01 3 = 0.52 4 = 0.87	*τ*_1_ = −2.07 *τ*_2_ = −2.50 *τ*_3_ = 1.49 *τ*_4_ = 3.08

Note. SD = standard deviation; Sep. = separation; Strat. = strata; Rel. = reliability; SE = standard error; Avg. Meas. = average measure; *τ* = Rasch–Andrich threshold; 0 = strongly disagree; students need far less support than described; 1 = disagree; students need less support than described; 2 = agree; 3 = disagree; students need more support than described; 4 = strongly disagree; students need far more support than described.

**Table 8:** Data fit to the MFRM model after collapsing categories.

	Facets								Rating Scale’s Categories	
	Items				Judges					
	SD	Separation	Strata	Reliability	*SD*	Separation	Strata	Reliability	Average Measure	*τ*
*Without extreme judges*	0.28	2.22	3.29	0.83	0.23	1.08	1.77	0.54	0 = −0.80 1 = −0.55 2 = −0.40 3 = −0.28	*τ*_1_ = −2.53 *τ*_2_ = 0.77 *τ*_3_ = 1.75
*All judges*	0.30	2.60	3.80	0.87	0.22	1.02	1.70	0.51	0 = −0.84 1 = −0.57 2 = −0.42 3 = −0.31	*τ*_1_ = −2.55 *τ*_2_ = 0.75 *τ*_3_ = 1.80

Note. SD = standard deviation; *τ* = Rasch–Andrich threshold; 0 = collapsed category representing less support than described; 1 = agree; 2 = disagree; students need more support than described; 3 = strongly disagree; students need far more support than described.

## Supplementary Materials

The following are available online at https://www.mdpi.com/1660-4601/18/2/764/s1. Table S1: B^W^_N_ and Bangdiwala’s agreement charts for the 366 indicators of the six age cohorts of the list of indicators.
