# Supplementary material for: Examining the Suitability of the List of Indicators Describing Age-Related Typical Support Needs
Source: Int J Environ Res Public Health. 2021 Jan 18;18(2):764. doi: 10.3390/ijerph18020764 (PMC7830960; doi:10.3390/ijerph18020764)
Supplement: Supplementary file 1 [file ijerph-18-00764-s001.pdf]

Supplementary material.

Table S1: B^{W}_{N} and Bangdiwala's agreement charts for the 366 indicators of the six age cohorts of the list of indicators.

**Age cohort 5-6 years**								
**Domain/Indicator**	**B^{W}_{N}**	**Chart**	**Domain/Indicator**	**B^{W}_{N}**	**Chart**	**Domain/Indicator**	**B^{W}_{N}**	**Chart**
HLA/01	0.90	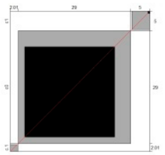	HLA/02	0.90	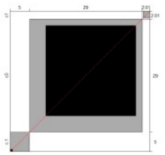	HLA/03	0.86	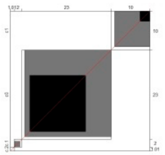
HLA/04	0.79	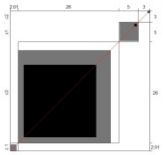	HLA/05	0.81	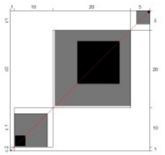	HLA/06	0.92	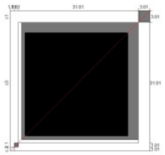
HLA/07	0.75	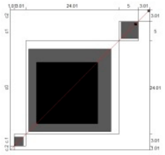	HLA/08	0.90	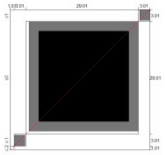	HLA/09	0.87	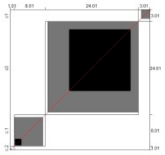
CNA/01	0.84	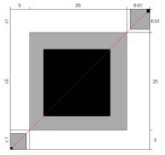	CNA/02	0.84	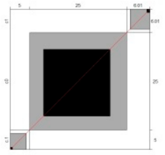	CNA/03	0.90	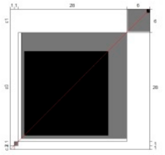
CNA/04	0.90	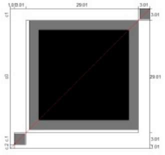	CNA/05	0.87	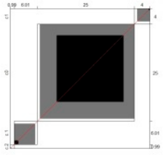	CNA/06	0.90	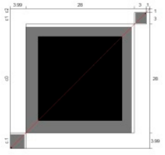

CNA/07	0.92	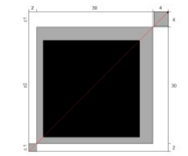	CNA/08	0.85	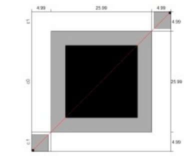			
SPA/01	0.85	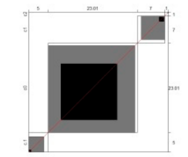	SPA/02	0.84	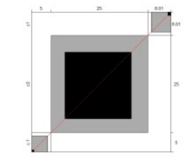	SPA/03	0.89	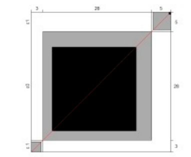
SPA/04	0.78	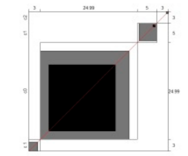	SPA/05	0.92	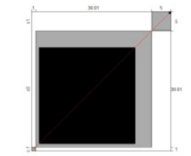	SPA/06	0.72	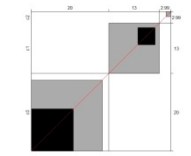
SPA/07	0.78	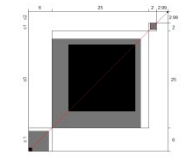	SPA/08	0.75	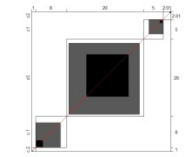	SPA/09	0.95	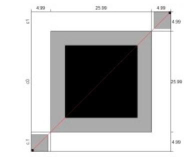
SLA/01	0.90	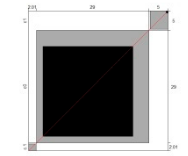	SLA/02	0.87	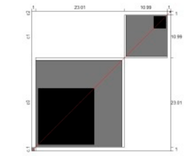	SLA/03	0.91	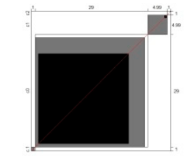
SLA/04	0.84	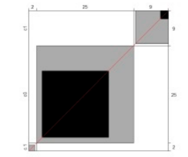	SLA/05	0.87	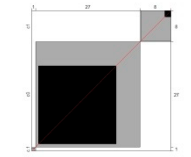	SLA/06	0.89	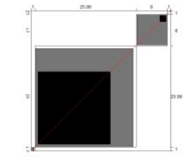

SLA/07	0.86	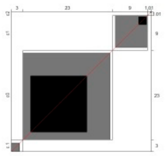	SLA/08	0.92	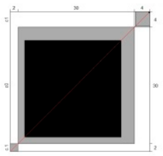	SLA/09	0.82	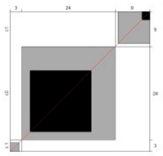
HSA/01	0.83	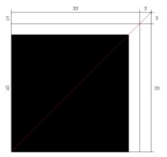	HSA/02	0.45	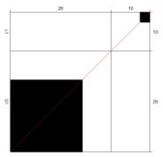	HSA/03	0.85	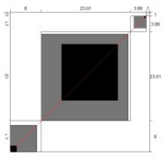
HSA/04	0.90	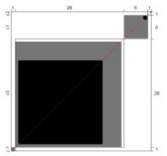	HSA/05	0.90	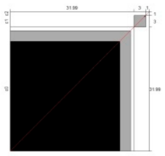	HSA/06	0.97	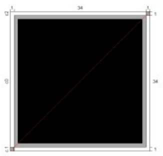
HSA/07	0.85	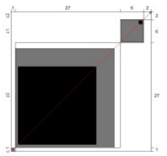	HSA/08	0.77	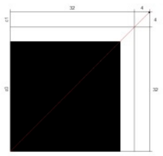			
SA/01	0.72	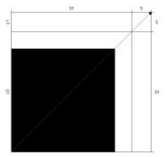	SA/02	0.87	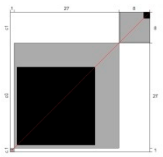	SA/03	0.89	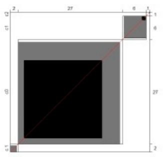
SA/04	0.91	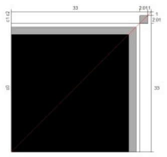	SA/05	0.94	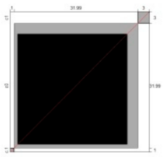	SA/06	0.90	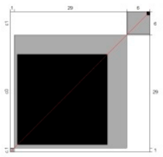

SA/07	0.89	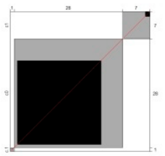	SA/08	0.89	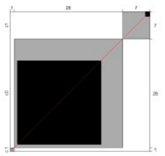	SA/09	0.80	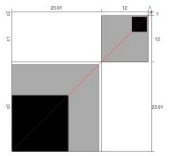
AA/01	0.96	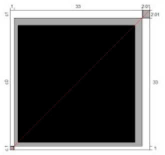	AA/02	0.55	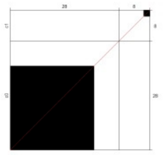	AA/03	0.89	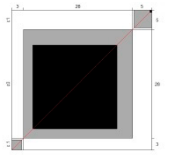
AA/04	0.87	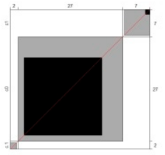	AA/05	0.92	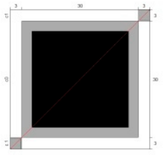	AA/06	0.83	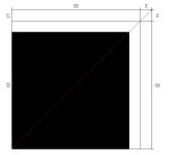
AA/07	0.87	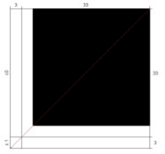	AA/08	0.45	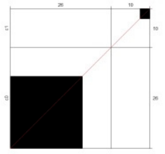	AA/09	0.86	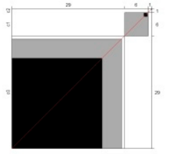

Age cohort 7-8 years								
**Domain/Indicator**	**B^{W}_{N}**	**Chart**	**Domain/Indicator**	**B^{W}_{N}**	**Chart**	**Domain/Indicator**	**B^{W}_{N}**	**Chart**
HLA/01	0.88	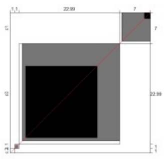	HLA/02	0.88	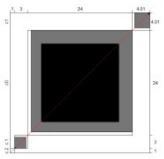	HLA/03	0.83	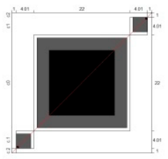
HLA/04	0.88	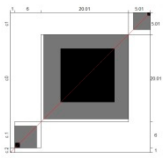	HLA/05	0.89	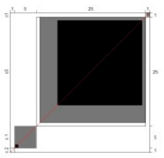	HLA/06	0.84	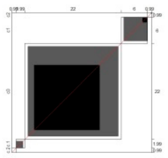
HLA/07	0.80	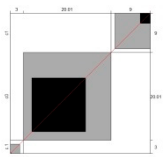	HLA/08	0.80	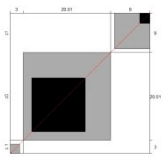	HLA/09	0.49	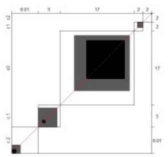
CNA/01	0.78	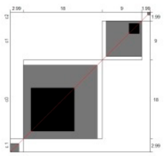	CNA/02	0.86	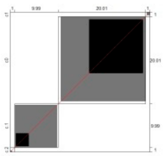	CNA/03	0.87	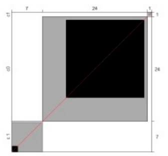
CNA/04	0.65	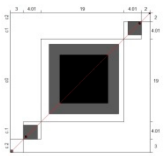	CNA/05	0.83	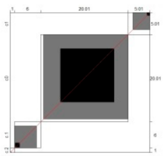	CNA/06	0.86	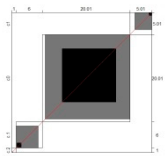

CNA/07	0.77	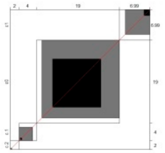	CNA/08	0.83	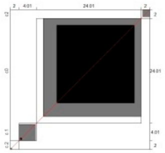			
SPA/01	0.85	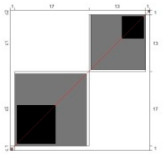	SPA/02	0.83	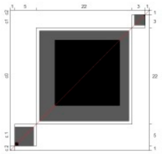	SPA/03	0.85	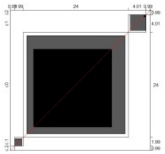
SPA/04	0.81	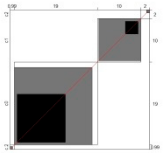	SPA/05	0.88	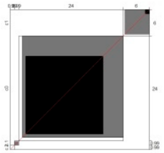	SPA/06	0.88	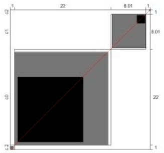
SPA/07	0.79	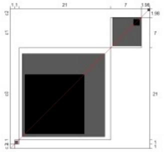	SPA/08	0.83	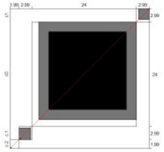	SPA/09	0.85	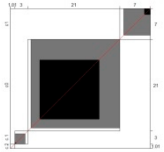
SLA/01	0.87	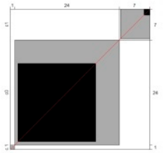	SLA/02	0.39	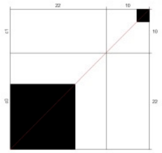	SLA/03	0.74	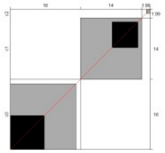
SLA/04	0.72	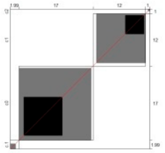	SLA/05	0.92	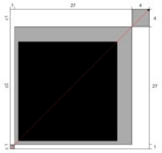	SLA/06	0.74	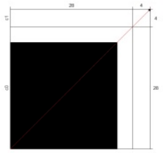

SLA/07	0.81	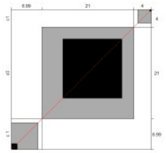	SLA/08	0.86	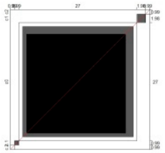	SLA/09	0.76	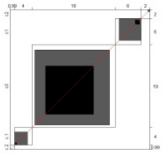
HSA/01	0.97	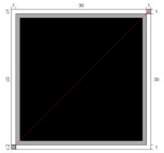	HSA/02	0.86	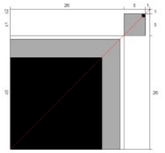	HSA/03	0.86	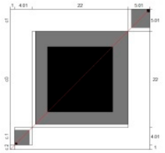
HSA/04	0.74	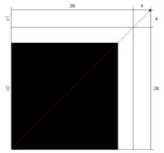	HSA/05	0.88	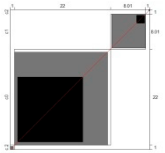	HSA/06	0.91	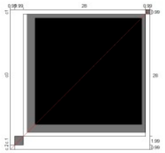
HSA/07	0.50	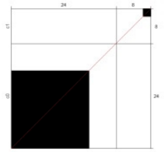	HSA/08	0.90	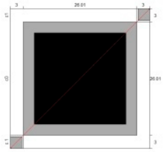			
SA/01	0.89	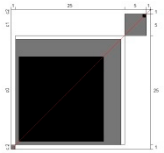	SA/02	0.82	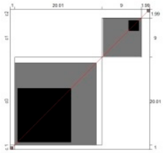	SA/03	0.62	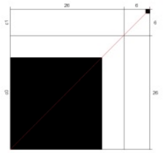
SA/04	0.79	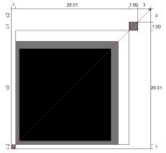	SA/05	0.87	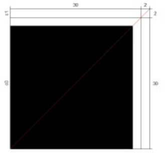	SA/06	0.92	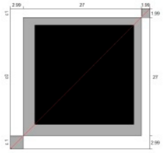

SA/07	0.95	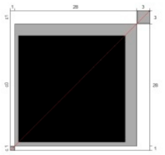	SA/08	0.87	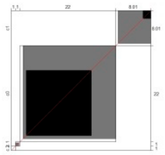	SA/09	0.80	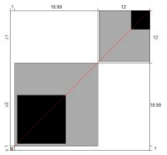
AA/01	0.96	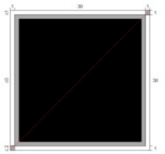	AA/02	0.87	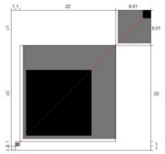	AA/03	0.89	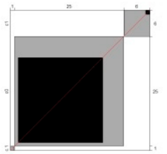
AA/04	0.90	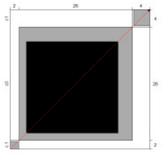	AA/05	0.62	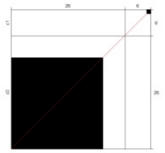	AA/06	0.87	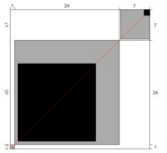
AA/07	0.94	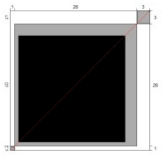	AA/08	0.76	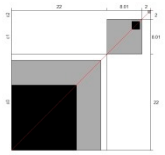	AA/09	0.86	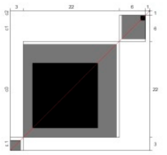

Age cohort 9-10 years								
**Domain/Indicator**	**B^{W}_{N}**	**Chart**	**Domain/Indicator**	**B^{W}_{N}**	**Chart**	**Domain/Indicator**	**B^{W}_{N}**	**Chart**
HLA/01	0.64	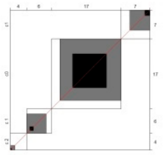	HLA/02	1	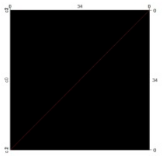	HLA/03	0.77	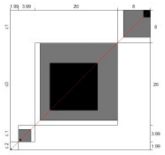
HLA/04	0.71	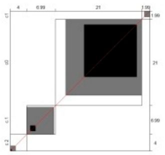	HLA/05	0.75	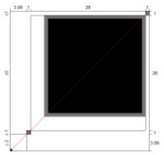	HLA/06	0.80	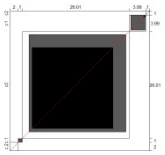
HLA/07	0.69	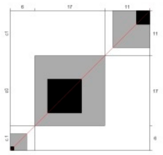	HLA/08	0.78	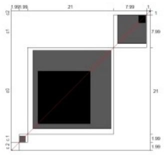	HLA/09	0.77	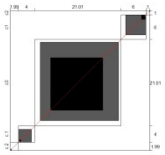
CNA/01	0.83	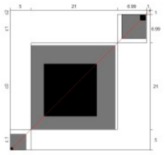	CNA/02	0.64	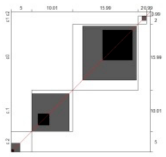	CNA/03	0.82	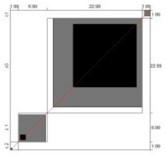
CNA/04	0.71	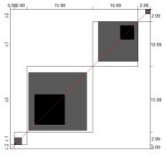	CNA/05	0.82	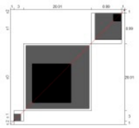	CNA/06	0.80	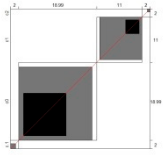

CNA/07	0.71	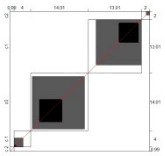	CNA/08	0.83	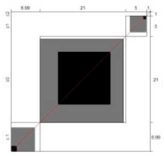			
SPA/01	0.77	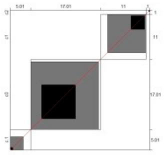	SPA/02	0.82	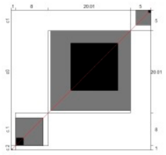	SPA/03	0.86	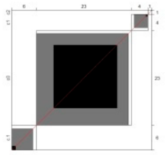
SPA/04	0.73	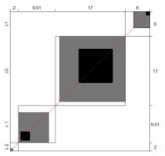	SPA/05	0.78	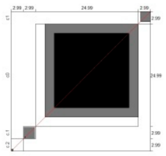	SPA/06	0.91	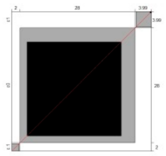
SPA/07	0.76	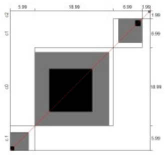	SPA/08	0.88	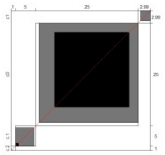	SPA/09	0.83	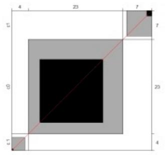
SLA/01	0.86	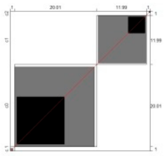	SLA/02	0.88	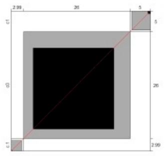	SLA/03	0.78	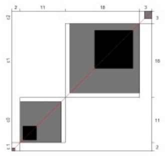
SLA/04	0.74	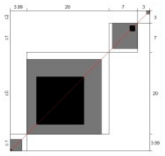	SLA/05	0.88	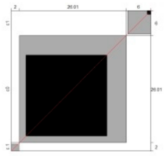	SLA/06	0.78	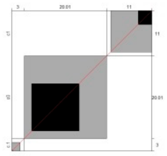

SLA/07	0.86	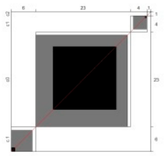	SLA/08	0.89	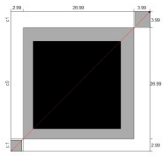	SLA/09	0.84	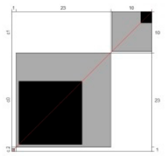
HSA/01	0.91	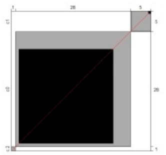	HSA/02	0.80	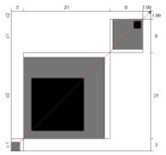	HSA/03	0.86	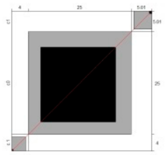
HSA/04	0.88	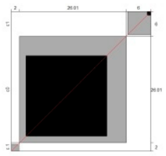	HSA/05	0.86	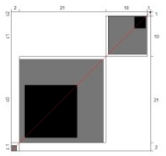	HSA/06	0.88	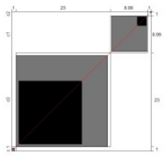
HSA/07	0.65	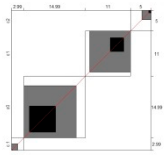	HSA/08	0.88	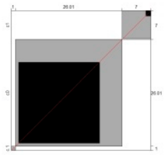			
SA/01	0.89	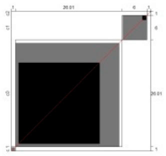	SA/02	0.87	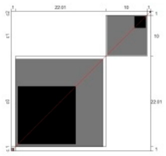	SA/03	0.88	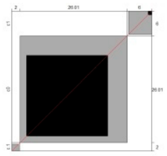
SA/04	0.81	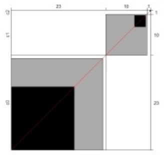	SA/05	0.88	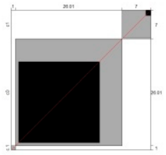	SA/06	0.95	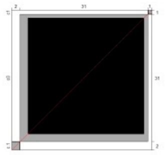

SA/07	0.70	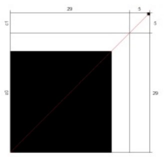	SA/08	0.88	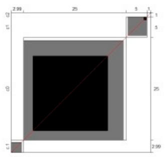	SA/09	0.80	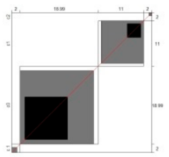
AA/01	0.89	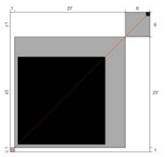	AA/02	0.78	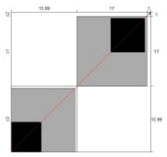	AA/03	0.83	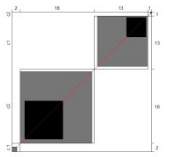
AA/04	0.87	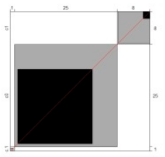	AA/05	0.78	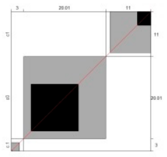	AA/06	0.77	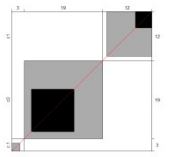
AA/07	0.82	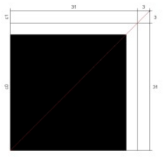	AA/08	0.81	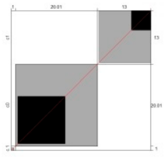	AA/09	0.76	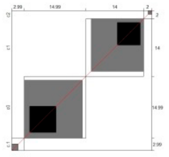

Age cohort 11-12 years								
**Domain/Indicator**	**B^W_N**	**Chart**	**Domain/Indicator**	**B^W_N**	**Chart**	**Domain/Indicator**	**B^W_N**	**Chart**
HLA/01	0.67	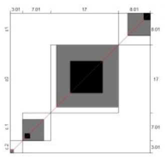	HLA/02	0.81	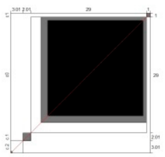	HLA/03	0.82	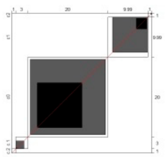
HLA/04	0.65	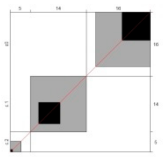	HLA/05	0.80	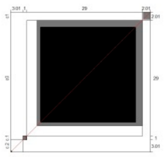	HLA/06	0.89	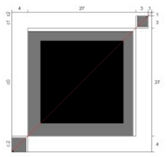
HLA/07	0.85	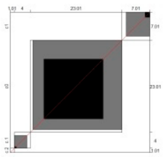	HLA/08	0.87	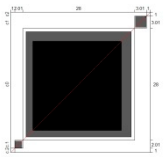	HLA/09	0.68	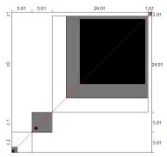
CNA/01	0.58	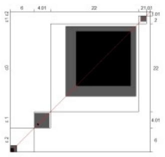	CNA/02	0.73	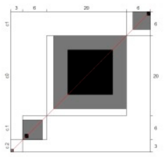	CNA/03	0.80	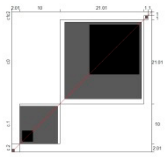
CNA/04	0.73	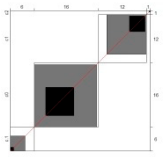	CNA/05	0.71	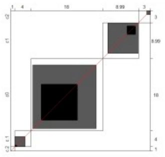	CNA/06	0.81	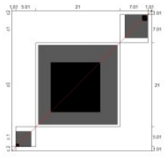

CNA/07	0.85	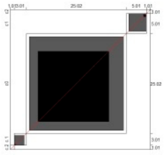	CNA/08	0.84	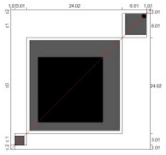			
SPA/01	0.85	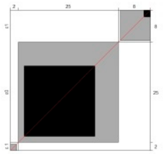	SPA/02	0.83	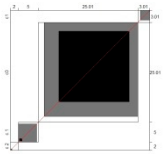	SPA/03	0.84	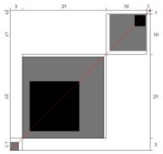
SPA/04	0.80	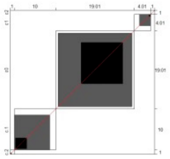	SPA/05	0.76	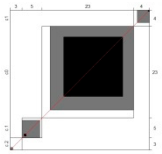	SPA/06	0.94	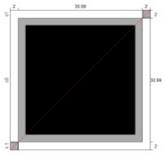
SPA/07	0.85	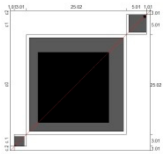	SPA/08	0.83	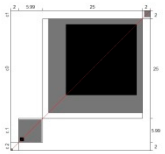	SPA/09	0.87	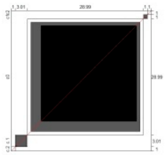
SLA/01	0.77	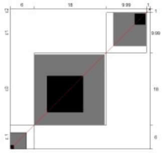	SLA/02	0.79	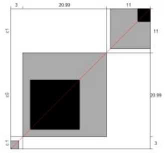	SLA/03	0.76	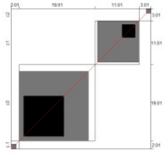
SLA/04	0.80	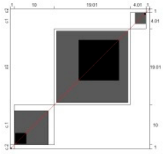	SLA/05	0.91	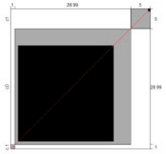	SLA/06	0.79	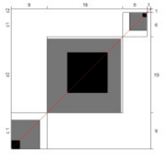

SLA/07	0.85	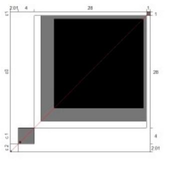	SLA/08	0.91	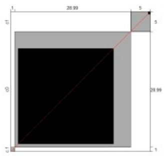	SLA/09	0.79	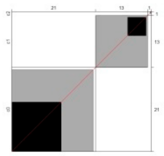
HSA/01	0.90	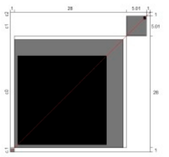	HSA/02	0.72	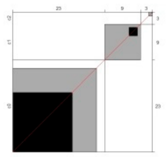	HSA/03	0.81	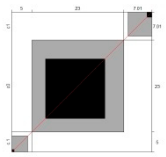
HSA/04	0.49	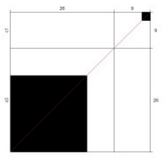	HSA/05	0.72	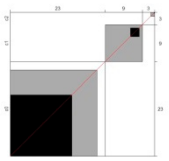	HSA/06	0.66	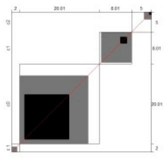
HSA/07	0.75	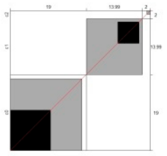	HSA/08	0.83	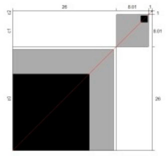			
SA/01	0.87	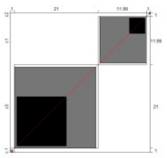	SA/02	0.82	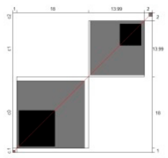	SA/03	0.82	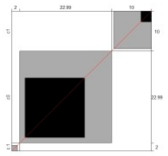

SA/04	0.86	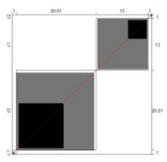	SA/05	0.86	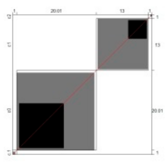	SA/06	0.86	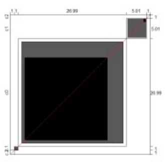
SA/07	0.85	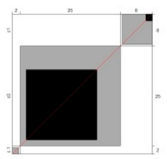	SA/08	0.87	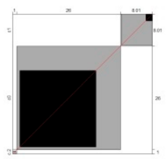	SA/09	0.84	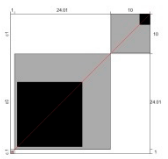
AA/01	0.85	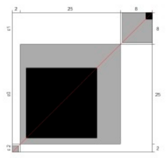	AA/02	0.83	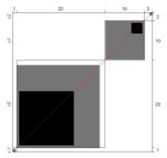	AA/03	0.87	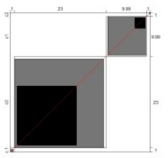
AA/04	0.87	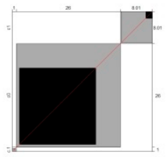	AA/05	0.80	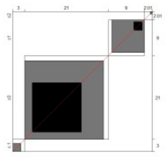	AA/06	0.76	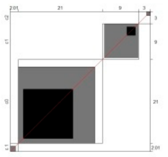
AA/07	0.92	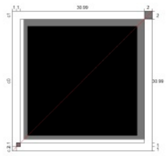	AA/08	0.88	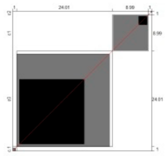	AA/09	0.87	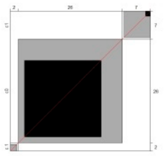

Age cohort 13-14 years								
**Domain/Indicator**	**B^{W}_{N}**	**Chart**	**Domain/Indicator**	**B^{W}_{N}**	**Chart**	**Domain/Indicator**	**B^{W}_{N}**	**Chart**
HLA/01	0.82	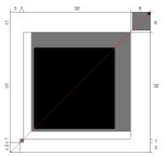	HLA/02	0.96	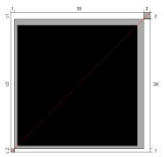	HLA/03	0.83	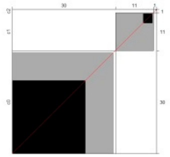
HLA/04	0.85	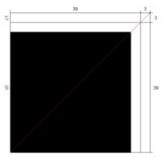	HLA/05	0.95	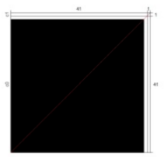	HLA/06	0.44	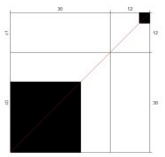
HLA/07	0.87	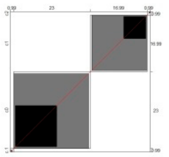	HLA/08	0.80	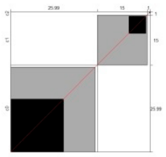	HLA/09	0.86	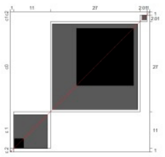
CNA/01	0.86	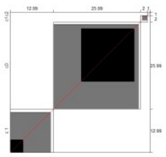	CNA/02	0.95	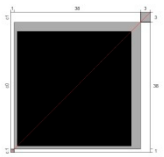	CNA/03	0.91	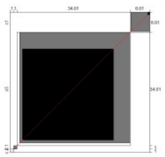
CNA/04	0.89	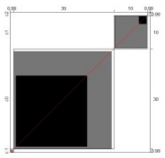	CNA/05	0.71	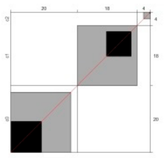	CNA/06	0.87	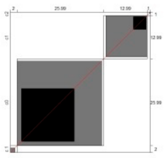

CNA/07	0.87	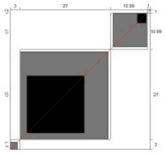	CNA/08	0.88	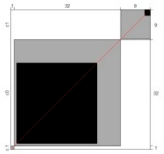			
SPA/01	0.86	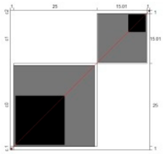	SPA/02	0.88	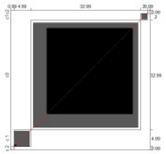	SPA/03	0.92	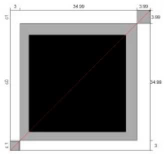
SPA/04	0.88	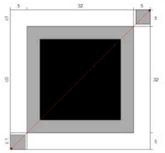	SPA/05	0.92	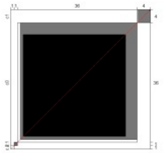	SPA/06	0.90	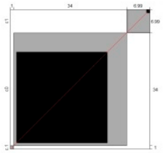
SPA/07	0.73	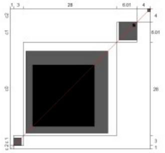	SPA/08	0.88	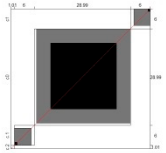	SPA/09	0.48	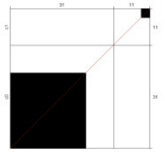
SLA/01	0.90	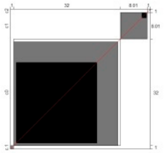	SLA/02	0.84	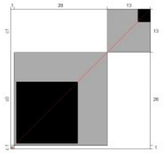	SLA/03	0.31	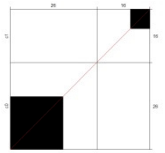
SLA/04	0.73	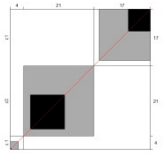	SLA/05	0.93	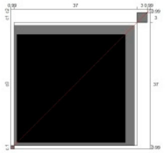	SLA/06	0.87	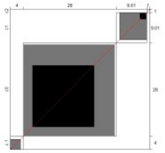

SLA/07	0.90	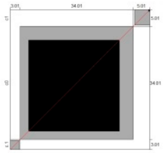	SLA/08	0.90	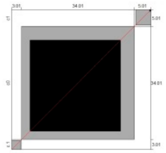	SLA/09	0.79	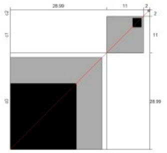
HSA/01	0.88	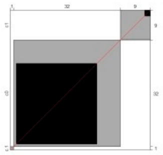	HSA/02	0.44	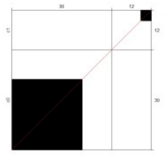	HSA/03	0.77	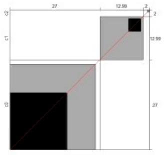
HSA/04	0.31	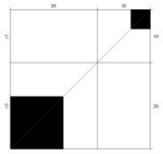	HSA/05	0.76	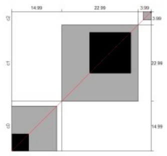	HSA/06	0.68	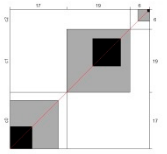
HSA/07	0.68	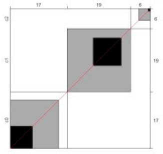	HSA/08	0.87	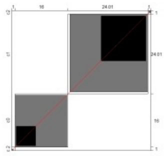			
SA/01	0.52	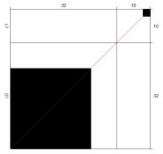	SA/02	0.81	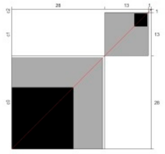	SA/03	0.57	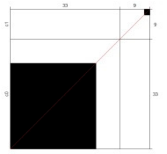
SA/04	0.73	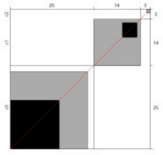	SA/05	0.83	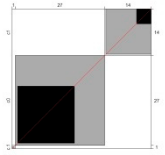	SA/06	0.89	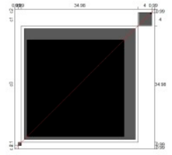

SA/07	0.87	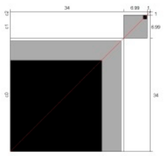	SA/08	0.74	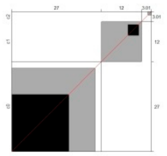	SA/09	0.77	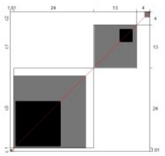
AA/01	0.57	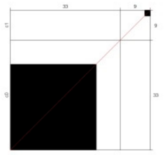	AA/02	0.84	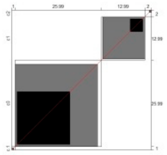	AA/03	0.78	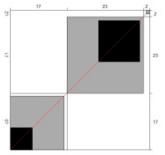
AA/04	0.75	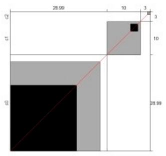	AA/05	0.83	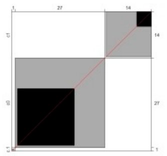	AA/06	0.86	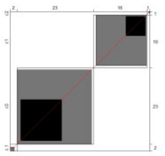
AA/07	0.91	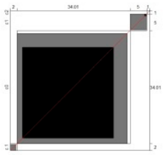	AA/08	0.88	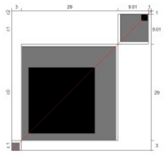	AA/09	0.84	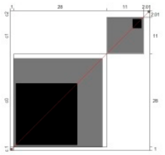

Age cohort 15-16 years								
**Domain/Indicator**	**B^{W}_{N}**	**Chart**	**Domain/Indicator**	**B^{W}_{N}**	**Chart**	**Domain/Indicator**	**B^{W}_{N}**	**Chart**
HLA/01	0.90	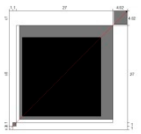	HLA/02	0.86	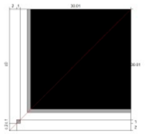	HLA/03	0.92	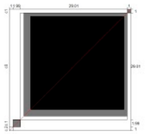
HLA/04	0.95	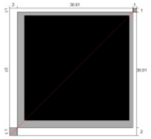	HLA/05	0.91	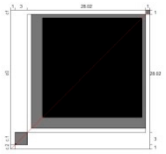	HLA/06	0.88	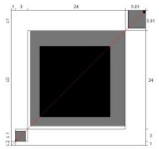
HLA/07	0.86	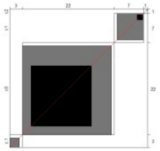	HLA/08	0.71	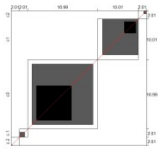	HLA/09	0.54	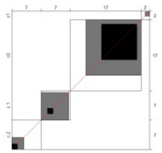
CNA/01	0.82	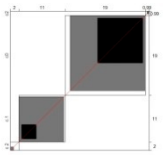	CNA/02	0.76	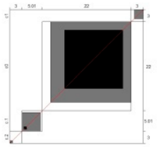	CNA/03	0.76	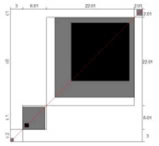
CNA/04	0.77	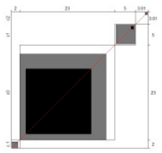	CNA/05	0.76	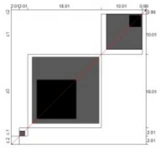	CNA/06	0.77	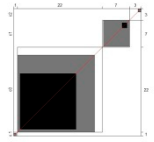

CNA/07	0.87	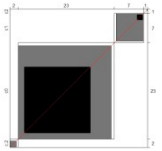	CNA/08	0.80	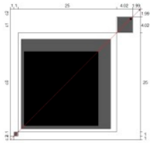			
SPA/01	0.82	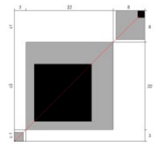	SPA/02	0.91	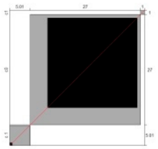	SPA/03	0.86	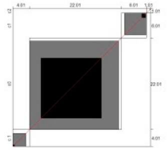
SPA/04	0.91	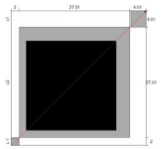	SPA/05	0.92	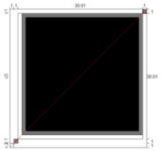	SPA/06	0.57	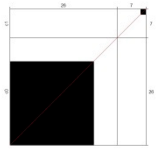
SPA/07	0.85	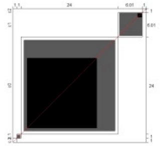	SPA/08	0.84	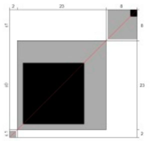	SPA/09	0.88	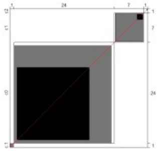
SLA/01	0.79	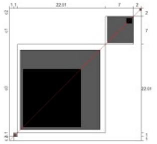	SLA/02	0.91	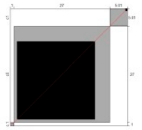	SLA/03	0.72	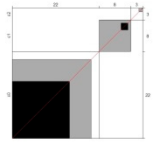
SLA/04	1	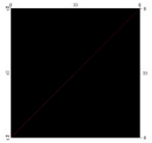	SLA/05	0.84	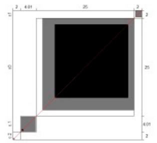	SLA/06	0.75	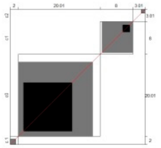

SLA/07	0.90	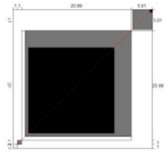	SLA/08	0.87	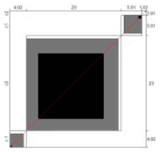	SLA/09	0.82	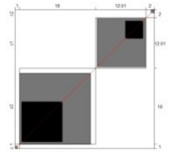
HSA/01	0.89	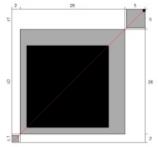	HSA/02	0.82	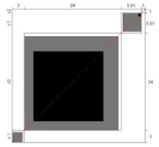	HSA/03	0.80	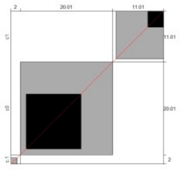
HSA/04	0.81	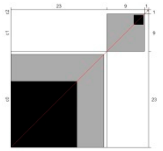	HSA/05	0.82	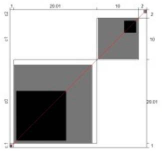	HSA/06	0.67	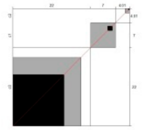
HSA/07	0.67	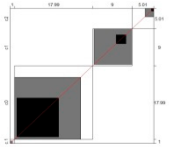	HSA/08	0.22	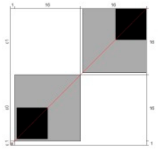			
SA/01	0.81	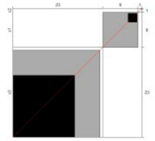	SA/02	0.75	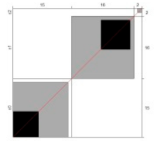	SA/03	0.71	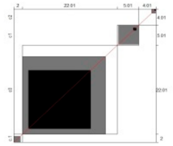
SA/04	0.76	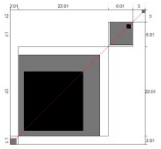	SA/05	0.84	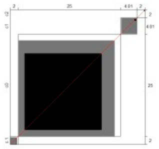	SA/06	0.85	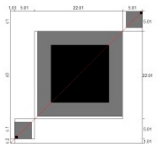

SA/07	0.86	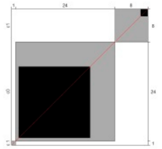	SA/08	0.82	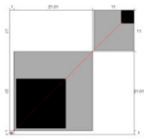	SA/09	0.72	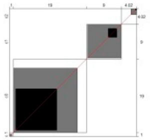
AA/01	0.86	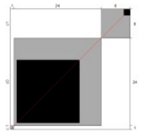	AA/02	0.66	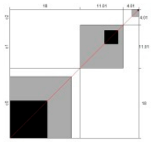	AA/03	0.74	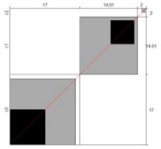
AA/04	0.85	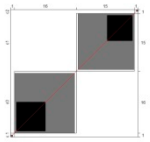	AA/05	0.79	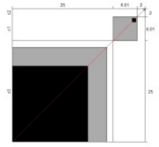	AA/06	0.87	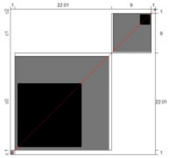
AA/07	0.82	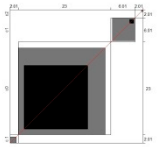	AA/08	0.78	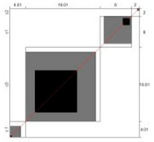	AA/09	0.71	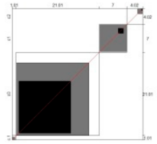

Note. B^{W}_{N} = Bangdiwala's weighted statistic; HLA = Home Life; CNA = Community and Neighborhood; SPA = School Participation; SLA = School Learning; HSA = Health and Safety; SA = Social Activities; AA = Advocacy.
